# The Interplay between Heat Shock Proteins and Cancer Pathogenesis: A Novel Strategy for Cancer Therapeutics

**DOI:** 10.3390/cancers16030638

**Published:** 2024-02-01

**Authors:** Prathap Somu, Sonali Mohanty, Nagaraj Basavegowda, Akhilesh Kumar Yadav, Subhankar Paul, Kwang-Hyun Baek

**Affiliations:** 1 Department of Biotechnology and Chemical Engineering, School of Civil & Chemical Engineering, Manipal University Jaipur, Dehmi Kalan, Jaipur 303007, India; prathaps1987@gmail.com; 2 Department of Biotechnology and Medical Engineering, National Institute of Technology, Rourkela 769008, India; sm22293@gmail.com; 3 Department of Biotechnology, Yeungnam University, Gyeongsan 38451, Republic of Korea; nagarajb2005@yahoo.co.in; 4 Department of Environmental Engineering and Management, Chaoyang University of Technology, Taichung 413310, Taiwan; yadavbasti@gmail.com; 5 Department of Bioengineering, Saveetha School of Engineering, Saveetha Institute of Medical and Technical Sciences, Chennai 602105, India

**Keywords:** heat shock proteins, molecular chaperones, cancer, HSP inhibitors, apoptosis, chemosensitizing agent

## Abstract

**Simple Summary:**

Heat shock proteins (HSPs) are extensively distributed throughout cells. They play a crucial role as molecular chaperones and regulate various cellular processes, such as metabolism, growth, differentiation, cell signaling, and programmed cell death. However, in cancers, HSPs are frequently overexpressed and associated with tumor advancement and metastasis, as well as in acquiring drug resistance against chemotherapeutic agents, leading to poor prognosis. Thus, the expression of HSPs can be modulated to imitate the cellular response against cancer cells by targeting the tumor microenvironment through different mechanisms. For instance, HSPs bind to antigens (peptides) associated with tumors, forming a complex that is easily recognized as an antigen-presenting cell (APC), leading to specific antitumor responses. This review summarizes and discusses recent advances, perspectives, and involvement of HSPs, including small and large HSPs, as well as the molecular mechanisms underlying tumor progression and metastasis. This study may offer new insights into the development of safer and more effective anticancer therapeutics.

**Abstract:**

Heat shock proteins (HSPs) are developmentally conserved families of protein found in both prokaryotic and eukaryotic organisms. HSPs are engaged in a diverse range of physiological processes, including molecular chaperone activity to assist the initial protein folding or promote the unfolding and refolding of misfolded intermediates to acquire the normal or native conformation and its translocation and prevent protein aggregation as well as in immunity, apoptosis, and autophagy. These molecular chaperonins are classified into various families according to their molecular size or weight, encompassing small HSPs (e.g., HSP10 and HSP27), HSP40, HSP60, HSP70, HSP90, and the category of large HSPs that include HSP100 and ClpB proteins. The overexpression of HSPs is induced to counteract cell stress at elevated levels in a variety of solid tumors, including anticancer chemotherapy, and is closely related to a worse prognosis and therapeutic resistance to cancer cells. HSPs are also involved in anti-apoptotic properties and are associated with processes of cancer progression and development, such as metastasis, invasion, and cell proliferation. This review outlines the previously mentioned HSPs and their significant involvement in diverse mechanisms of tumor advancement and metastasis, as well as their contribution to identifying potential targets for therapeutic interventions.

## 1. Introduction

Heat shock proteins (HSPs) are ubiquitous polypeptide proteins found mostly in every cell from prokaryotes to eukaryotes [1]. HSPs are essential cellular components contributing to the maintenance of cellular protein homeostasis and internal conditions under both moderate and damaged growth conditions [2,3]. It is commonly known that HSPs are involved in a variety of cellular functions, including protein synthesis, folding and assembly, translocation, conformational maintenance, and degradation during cellular processes. These proteins also contribute to the normal growth and development of cells [4,5]. They play essential roles in a myriad of cellular activities: assist in protein synthesis, prevent aggregation of unfolded or misfolded proteins that aid in proper folding and assembly to form functional structures, aid in the translocation of proteins for their proper localization within the cell, maintain proper conformation, thus preventing proteins to acquire potentially harmful structures, help in the degradation of misfolded proteins through proteolytic pathways such as the ubiquitin-proteasome system, and finally assist in the regular processes of cellular growth and development by playing regulatory roles in signaling pathways that control cell proliferation and differentiation. HSPs also have a crucial role in activating client proteins within cells [6]. They have also been found to help in membrane stabilization and protein refolding under stressed conditions [7]. They also help to eliminate damaged or old cells under stress conditions to restore cellular homeostasis [8,9].

Cellular stress includes elevated temperature, reduced oxygen availability, ischemic conditions, and exposure to harmful substances, pathogens, ultraviolet (UV) radiation, or inflammatory signaling molecules. Exposure of organisms to pathophysiological, metabolic, or environmental stress conditions leads to the selective upregulation of HSPs as a natural cellular defense response [10]. This elevated expression of HSPs helps cells resist and thus acts via cytoprotective mechanisms [11].

## 2. Major HSPs and Their Role in the Various Biological Pathways of Cells

Classified by their molecular weights, HSPs fall into six primary families: HSPH denoted as HSP110, HSPC denoted as HSP90, HSPA denoted as HSP70, DNAJ denoted as HSP40, HSPB- small HSPs, and chaperonins, HSP60. The HSPB gene family generates small HSPs, including 11 ubiquitous molecular chaperones that function independently of ATP. The small HSPs have a size that varies between 12 and 43 kDa. Their size is determined by a β-sandwich structure of the α-crystallin domain, which is conserved. This structure is surrounded by N- and C-terminal sections that can vary greatly [12]. Several high-molecular-weight HSPs are well documented as ATP-dependent chaperones; that is, their function depends on ATPase activity [13]. For example, the HSP40 family is encoded by DNAJ genes and, being the largest among the various HSPs, has 49 members. The chaperonin family of HSP60 in humans consists of 14 members encoded by CCT, HSPD/E, and various other genes [11].

Members of the HSP70 family encode 13 HSPA genes. HSPC contains five homologous members of the HSP90 family. Large HSP families have two members, HSP110 and GRP170 (glucose-regulated protein 170), which are encoded by the HSPH gene family [11,14]. HSP expression is regulated by multiple factors, including cellular stress factors, generally known as the heat shock response (HSR) [15], and is regulated by heat shock factors (HSFs) at the transcriptional level [16]. In vertebrates, HSFs such as HSF1, HSF2, HSF3, HSF4, and HSFY mutually utilize identical structures, with the most highly conserved N-terminal helix-turn-helix DNA-binding domain and C-terminal transactivation domain [17]. Heat Shock Factor 1, initially identified as the primary regulator of HSR and the production of HSP genes, functions via cis-regulatory elements located in the promoter region upstream of HSP genes, which are known as heat shock elements (HSEs) [18]. In contrast to the traditional HSR, certain HSP members, such as HSC70, GRP78, MTP70, and HSP90β, and almost none of the types of HSPs require stress or heat shock for their expression; however, they are constitutively expressed under normal conditions [19]. Table 1 describes the various types of HSP families and their relevant members, along with their cellular locations and proposed functions.

Moreover, HSP expression may be constitutive (cognate) or inductively regulated. Hence, the HSP family can be classified based on their expression as constitutive or inducible HSPs. For instance, HSP73 exhibits continuous expression, whereas the closely related protein HSP72 is responsive to stress for its induction. Both HSP72 and HSP73 function as molecular chaperones, engaging in brief interactions with newly formed polypeptides [33]. A study indicated the dispersion patterns of the constant HSC70 (heat shock cognate 70) and stress triggered HSP70 within the 70 kDa HSP category. The distribution of HSP70s is related to the metabolic needs of the rat eye, which are required for the detection and absorption of light [34]. HSP70 expression is usually strongly induced by stress, but immune cells express these proteins under normal conditions. However, HSC70 is constitutively expressed in mammalian cells [34]. First, the trimerization of HSF occurs before the affinity of the heat shock element (HSE) to the DNA. Phosphorylation triggered by stress is pivotal in regulating the activation and interaction of HSF1 with DNA. The association between HSBP1 and HSF1 monomers hinders the activation of HSF1 [35]. These results suggest that HSP70 can trigger the transition from a fully activated HSF trimer to an inactive monomer [36]. This represents a critical element in reducing the transcriptional response to heat shock. In summary, stress induces HSP70 expression, constitutive expression of HSC70 is observed, and the regulation of HSP expression involves HSFs, trimerization, and stress-dependent phosphorylation, with HSP70 influencing the transition of active HSF trimers to inactive monomers.

Recent studies have reported that molecular chaperones are essential for the translocation of proteins from one cellular compartment to another. For example, mitochondria and chloroplasts import over 95% of their protein constituents from the cytosol, which is further transported into a particular cell organelle via a specific protein import machinery called ATP-driven HSP70 (cpHSP70) [37]. In another study, Sin et al. reported that sHSP20 (small heat shock protein 20) is essential for the nuclear translocation of PKD1 (protein kinase D1) [38].

**Figure 1:**
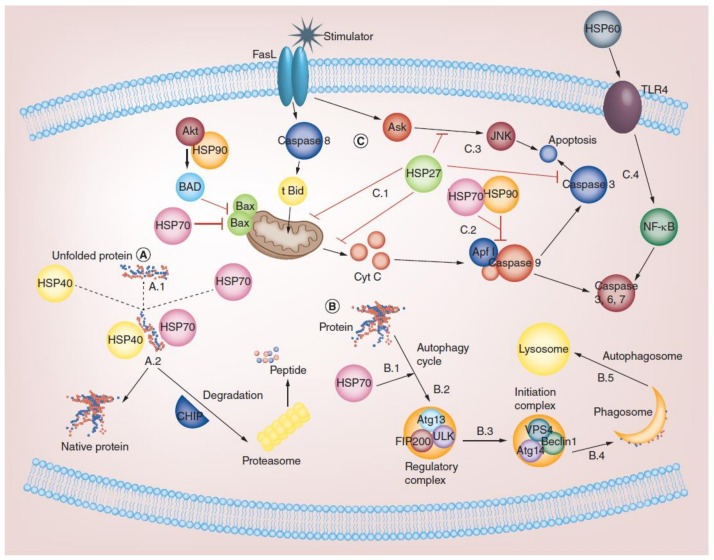
Role of HSP in biochemical functions of cells. (A) Chaperone activity: misfolded proteins that accumulate in the cytosol bind to HSPs to maintain homeostatic balance (A.1). For example, the HSP70/HSP40 complex guides the degradation of misfolded proteins into short peptides via proteasome (A.2). (B) Role of HSP in the autophagy pathway: the autophagy cycle begins by binding HSP70 to the protein (B.1). Regulation of Atg13, FIP200, and ULK1 complex induced by HSPs (B.2). The initiation of the VPS4, Atg14, and Beclin1 complexes is regulated by HSPs (B.3). Ultimately, phagosomes are formed using phagosomal markers (B.4). Finally, autophagosomes or amphisomes fuse with lysosomes (B.5). (C) Role of HSP in different apoptotic pathways: HSPs help in modulating pro-apoptotic signals through FasL at the mitochondrial and post-mitochondrial levels. HSP70 and HSP27 appear to suppress the release of mitochondrial pro-apoptotic proteins by activating Bax and tBid (truncated Bid), respectively (C.1). HSP27, HSP70, and HSP90 can interact with and recruit Apaf-1 (apoptosis protease-activating factor 1) by directly sequestering cytochrome C to prevent apoptosome oligomerization and activation (C.2). HSP27 can also inhibit apoptosis via inactivation of caspase 3 and ASK (apoptosis signal-regulated kinase) (C.3). Furthermore, HSP60 and TLR-4 interactions mediate the NF-kappaB (NF-κB) signaling pathway, resulting in the activation of caspases 3, 6, and 7 and DNase (C.4). Reprinted (adapted) with permission from Ref. [44]. Copyright 2019, Future Medicine Ltd., London, UK.

HSPs participate in various cellular functions such as protein folding, acting as chaperones, regulating apoptosis, managing autophagy, and contributing to immunity. Broadly, these proteins safeguard cells against stressful situations, including elevated temperatures. Additionally, diverse functions of HSPs have been associated with their role in the immune system. These encompass intracellular roles, such as antigen expression and presentation facilitated by innate immunity, as well as extracellular functions, such as participating in tumor immunosurveillance and autoimmunity [39]. Extracellular HSPs also occur in extracellular sites, plasma membrane, cytosol, extracellular vesicles, and biological fluids. Extracellular HSPs interact with the immune system and serve as regulators and stimulants [39]. Based on their location in intracellular or extracellular sites, HSPs have been demonstrated to have dual roles, as reported in multiple studies. Extracellular HSP90 (for example HSP90α) is located on the cell surface of tumors facing the extracellular space and plays an important role in tumor metastasis [40]. Similarly, extracellular HSP70 has been detected in the medium of antigen-presenting cells (APCs), inducing T-cell activation. Extracellular HSP60 has been identified in the plasma of some individuals, but its source remains unknown [41]. Extracellular HSPs such as HSP27, αBC, and HSP20 are secreted into the extracellular medium via non-classical pathways and play important roles in cell-to-cell communication, cell signaling, immunity, stress tolerance, inflammation, and anti-apoptotic activities [42]. Internally located HSPs perform a vital function in safeguarding cells through various mechanisms, providing essential cytoprotection. HSP27 and various molecular machinery involved in programmed cell death are directly involved in its interaction with HSP70 [43]. The biological functions of different HSPs in various cellular processes are shown in Figure 1.

## 3. HSPs and Cancer

The rapid growth of cancer cells demands constant protein synthesis to support replication, leading to significant alterations in signal transduction pathways and metabolic stress. As a result, cancer cells are more protein-dependent. Hence, cancer cells are increasingly dependent on proteins, such as stress-inducible HSPs with molecular chaperone activity, for protein folding to the native form. Furthermore, the cytoprotective functions of HSPs are essential for the maintenance and survival of cancer cells. Undoubtedly, HSP27, HSP70, and HSP90 have been associated with cancer cells, which also indicates that HSPs control cellular processes and functions during carcinogenesis [14].

Numerous studies have documented an increase in the expression levels of HSPs in cancer cells when exposed to different stimuli, such as chemotherapy, inhibition of tyrosine kinases, oxidative stress, elevated temperature (hyperthermia), and activation of the Fas (Apo-1/CD95) apoptotic receptor [16]. This confirmed a notable interaction between the expression levels of HSPs and the resistance of cancer cells during chemotherapy. Various studies have shown that inhibition or depletion of HSPs significantly reduces tumor size and hence cancer progression, as in the case of HSP70. Therefore, targeting different HSPs has been proposed as a potential innovative approach for cancer therapy.

### 3.1. Role of HSP27 in Cancer Development and Progression

#### 3.1.1. Structure, Function, and Interaction of HSP27

**Figure 2:**
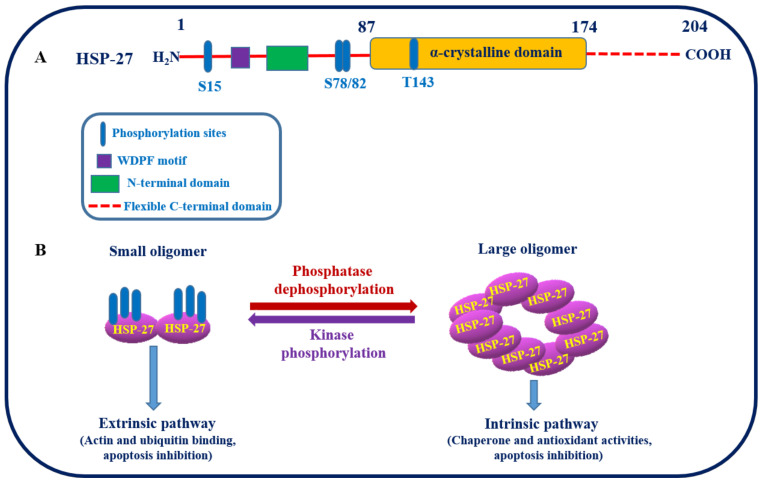
(**A**) Structure of HSP27 and its putative phosphorylation sites. (**B**) Schematic representation showing the two different conformational states of HSP27 induced by phosphorylation, that is, large oligomers when unphosphorylated and small oligomers when phosphorylation is catalyzed by MAPKAPK 2/3 kinase at specific serine residues of HSP27. These conformational, structural, and functional changes actively contribute to the maintenance of cellular proteostasis [49].

HSP27, an important chaperone, belongs to the sHSP family with a common highly conserved alpha-crystallin domain, as shown in Figure 2A [45]. It plays an essential role in cell proliferation, invasion, metastasis, differentiation, and death. The molecular chaperone HSP27 is ubiquitously distributed in the body and is regulated by phosphorylation at three specific serine residues (Ser82, Ser78, and Ser15), resulting in the formation of an oligomer. The process of phosphorylation described is reversible and primarily facilitated by the enzyme mitogen-activated protein kinase-activated protein kinase 2/3 (MAPKAPK 2/3 kinase). The activation of these MAPKAPK occurs when upstream kinases phosphorylate the p38 mitogen-activated protein kinase (Figure 2B) [46]. The oligomeric status of HSP27 regulates its function as a molecular chaperone [47]. For instance, dephosphorylation provokes large oligomer formation and shows intrinsic chaperonin activity, whereas phosphorylation favors small oligomer formation, which binds to the microfilaments to stabilize HSP27 as an extrinsic function, as shown in Figure 2B [48]. However, under in vivo conditions, the oligomerization of HSP27 is entangled with cell–cell interactions, and the phosphorylation status is independent. Client proteins involved in various stages of the apoptotic pathway, such as p53, NF-kB, and HSP27, control apoptosis by regulating Akt. Overexpression of HSP27 reduces apoptotic cell death in response to different cellular stressors, such as cytotoxic drugs, hyperthermia, and oxidative stress. The anti-apoptotic functions of HSP27 have also been identified through the formation of large oligomers and their tumorigenic properties.

#### 3.1.2. HSP27 as Target for Cancers Therapeutics

In several studies, HSP27 was found to be overexpressed, playing critical roles in tumorigenesis, metastasis, and invasiveness, as well as in the poor prognosis of various cancers. Moreover, the phosphorylation patterns of HSP27 in cancer cells are unique and align with those in primary non-transformed cells [50]. Hence, the distinct phosphorylation patterns of HSP27 may be utilized as a useful tumor biomarker [51]. A high level of HSP27 in the tissues of meningioma patients, confirmed by immunohistochemical and Western blot studies, correlated with poor prognostic factors in cancer patients [52]. The overexpression and heat shock accumulation of HSP27 are associated with several characteristics of carcinogenesis, including multi-drug resistance, increased cytoprotection, and apoptosis inhibition with several pro-apoptotic proteins [53,54]. Thus, HSP27 is considered a promising therapeutic target in cancers because of its important role in drug resistance and tumor progression [55,56].

#### 3.1.3. HSP27 as a Therapeutic Target

**Figure 3:**
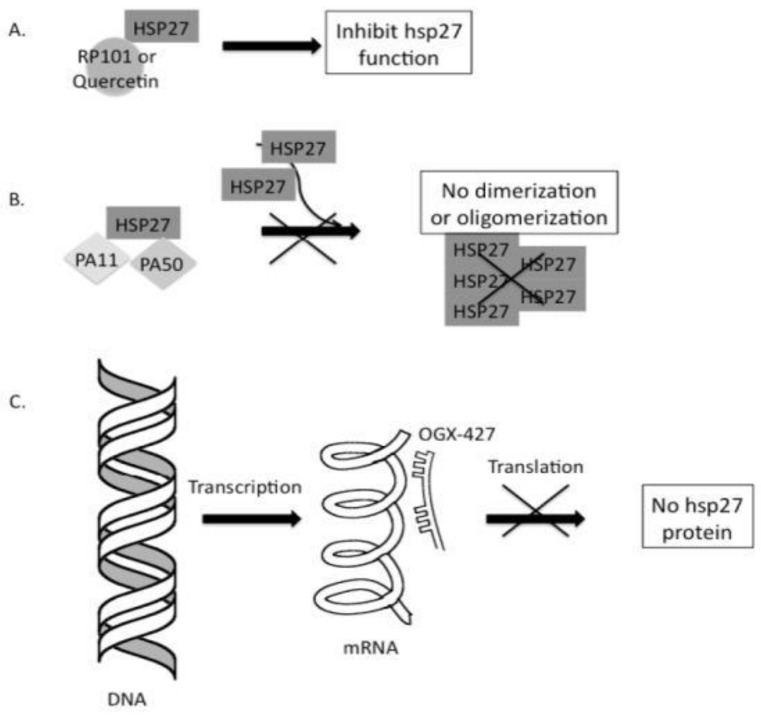
Strategies targeting cancer cells based on HSP27 inhibition. (**A**) Small molecules, such as quercetin and RP101 (brivudine), can directly bind to HSP27 and suppress its function. (**B**) Peptide aptamers such as PA11 and PA50 can specifically bind to HSP27, inhibiting HSP27 dimerization or oligomerization, thereby disabling its function. (**C**) The final strategy applies Apatorsen (OGX-427), an antisense oligonucleotide that binds to HSP27 mRNA and blocks HSP27 translation, thereby HSP 27 protein production inhibited. Consequently, the amount of HSP27 is reduced, causing cell death, which protects the cell through its anti-apoptotic activity. Reprinted (adapted) with permission from Ref. [61]. Copyright 2013, Elsevier.

Three distinct strategies for cancer therapies based on HSP27, as shown in Figure 3, are described as follows:

❖ First, the inhibition of HSP27 is associated with the binding of small molecules [57,58].
❖ Second, protein aptamers are intended to bind to certain proteins of interest, consequently disrupting and inhibiting their functions and preventing dimerization or oligomerization [59].
❖ Finally, antisense oligonucleotide (ASO) binds to HSP27 mRNA and inhibits HSP27 translation [60].

HSP27 inhibitors, such as quercetin and RP101, are two important molecules that have already been studied. RP101 (Brivudine) also known as bromovinyldeoxyuridine (BVDU) is an antiviral nucleoside that binds with HSP27 via binding π-stacking with Phe-29 and Phe-33 of HSP27 to inhibit its function (Figure 3A) [58,62]. When RP101 binds to HSP27, there is less binding of HSP27 to pro-caspase3, cytochrome C, and Akt1, which affects apoptosis [58,63]. Hence, the development of RP101 as a cancer drug is a unique approach to enhance chemosensitivity and prevent chemoresistance. In clinical studies involving stage 3 and 4 pancreatic cancer patients, the median overall survival rate increased by more than 8.5 months due to the RP101vs historical control group (NCT00550004) [58]. Additionally, during a phase II clinical trial targeting pancreatic cancer, the combination of RP101 with gemcitabine resulted in an increase in survival period by around 2.17 months. Despite the termination of the clinical study due to adverse side effects of gemcitabine, the RP101-based trial reported no such adverse effects. Consequently, the advancement of second-generation RP101 candidates is presently in the developmental stages.

Quercetin is a bioflavonoid widely distributed in ferns. This compound is also a popular natural compound with anticancer activity via HSF1 (heat shock transcriptional factor1)-dependent HSPs in several cancer cell lines [64]. Most non-small-cell lung cancer (NSCLC) cell lines, such as A549, have been reported to inhibit HSP-27, thereby reducing cell survival. Furthermore, quercetin also serves as a chemosensitizer when used in combination with chemotherapeutics, such as cisplatin or gemcitabine, as it exhibits more potent cytotoxic activity in A549 cells than when applied alone [65]. However, the exact mechanism of action of quercetin is not adequately understood, and a recent study showed that the inhibition of cellular expression of CK2 (casein kinase 2) by quercetin facilitates the proteasome-mediated degradation of HSP27 [66,67]. Casein kinase 2 plays an essential role in increasing the stability of HSP27 protein [68], indicating that quercetin acts as an important regulator of HSP27 protein stability by inhibiting CK2. It is a widely recognized natural compound that exhibits anticancer effects through the regulation of heat shock transcriptional factor 1 (HSF1)-dependent HSPs in numerous cancer cell lines [18]. In several NSCLC cell lines, including A549, it has been observed that HSP27 inhibition occurs, leading to a decrease in cell viability.

The second approach involves the application of specific peptides that inhibit HSP27 anti-apoptotic activity by binding to a particular protein domain (Figure 3B) [69]. Currently, PA11 and PA50 are two important peptide aptamers that specifically bind to HSP-27, thereby interfering with its oligomerization or dimerization, and disrupting its function. PA11 inhibits HSP27 oligomerization, which ultimately perturbs early cellular proteostasis and induces proteotoxic stress and cell death ensues [59]. The mechanism of PA50 is to inhibit the dimerization of HSP27. By inhibiting HSP27 dimerization, PA50 interrupts HSP27 to participate in cell-signaling events that are essential for cell survival.

Moreover, like molecular inhibitors, peptide aptamers are not capable on their own but function as chemo-sensitizing agents in combination with other therapies to sensitize cancers. For instance, both PA11 and PA50 accelerate the cellular radiosensitivity of HNSCC (head and neck squamous cell carcinoma) cell lines by 47% and 32%, respectively, against control. Although peptide aptamers have demonstrated promising results in preclinical models and trials for cancer therapy, they encounter specific limitations. These constraints include size restrictions on the studied proteins, challenges in addressing membrane protein complexes, and difficulties in operating in an RNase-free environment. To address these issues, overcoming obstacles and enhancing the understanding of the structure–function relationship of HSP27 is crucial, leading to the discovery of new anticancer drugs.

The third approach involves the application of second-generation antisense oligonucleotides (ASOs) that target HSP27 mRNA. The sequence of Apatorsen or OGX-427 (OncoGenex Pharmaceuticals, Bothell, WA, USA) is an antisense oligonucleotide that inhibits the expression of HSP27 (Figure 3C) by preventing HSP27 mRNA translation through binding to the initiation site (5′-GGGACGCGGCGCTCGGTCAT-3′) [70]. In a xenograft model of prostate cancer (PC-3), the co-administration of OGX-427 and chloroquine resulted in a 50% reduction in tumor volume after a seven-week treatment period, compared to the effect observed with chloroquine treatment alone [71].

Inhibition of HSP27 significantly enhanced radiation-induced cell death and apoptosis and promoted Akt inactivation. In SQ20B-cancer-cell-bearing mice (radiation-resistant lung cancer), knockdown of HSP27 using OGX-427 enhanced the efficiency of radiation therapy by increasing the cytotoxic efficacy and decreasing glutathione antioxidant defenses and cell survival [72]. Radiation and OGX-427 decreased angiogenesis, which was related to a reduction in Akt pathway activity. This combination treatment also enhances cell survival and antitumor effects in SQ20B-tumor-bearing mice, with no effect on acute or delayed tumor growth [72]. Furthermore, a clinical study demonstrated that the survival rate of patients with metastatic pancreatic cancer and the duration of progression-free survival were extended when standard chemotherapy was combined with OGX-427.

In summary, HSP27 plays an important role in cancer treatment and has become a novel therapeutic target. However, HSP27, with its greater structural complexity, has become a significant challenge in the search and design of therapeutic molecules that can be easily neutralized. Both the large and small oligomers of HSP27 are in dynamic equilibrium, which is regulated by HSP27 phosphorylation and functions in its oligomeric form. Therefore, it is preferable to target only the phosphorylated or unphosphorylated forms of HSP27 using various molecules. Moreover, HSP27 is involved in the prevention of protein aggregation, thereby inhibiting several neurodegenerative diseases. Hence, HSP27 may be considered for the prevention of neurodegenerative diseases along with its use as a target in cancer therapy.

### 3.2. Role of HSP40 in Cancer Progression and Potential Therapeutic Target

#### 3.2.1. HSP40 Structural Studies and Functional Implications

The HSP40 family plays a crucial role in the translation, folding, refolding, translocation, degradation, and assembly/disassembly of proteins. The DNAJ protein family encodes 49 proteins in humans and is primarily categorized into three subtypes, types I, II, and III (also indicated as DNAJA, DNAJB, and DNAJC, respectively), depending on the presence and location of the domain structure [73]. Type I HSP40 (DNAJA) comprises four protein domains: an N-terminal JD (J-domain), a GF-motif (a glycine/phenylalanine-rich region), a Cys-repeat (a cysteine repeat), and a largely uncharacterized C-terminus [74]. Type II (DNAJB) comprises 13 distinct members, characterized by the absence of the Cys-repeat region and an elongated GF-motif region. In contrast, Type III (DNAJC) encompasses 32 members, exhibiting substantial distinctions from both Type I and Type II DNAJs. Notably, Type III lacks both GF-motif and Cys-repeat regions, and the J-domain may be positioned anywhere within the protein domain. The structure of the J-domain consists of approximately 70 amino acids folded into four α-helices (helices I–IV), with a loop region between helices II and III containing a highly conserved tripeptide, the HPD (His-Pro-Asp) motif [75]. The helical wheel representation of helix II is crucial for its interactions with the ATPase domain of HSP70 [76].

DNAJ stabilizes the interaction between HSP70 and its substrates. The highly conserved HPD motif of the J-domain plays an essential role in HSP40/HSP70 interactions, and HSP40 stimulates HSP70 ATPase activity [77]. The ATPase domain of HSP70 results in ATP hydrolysis to convert HSP70 into an ADP-bound state, thus maintaining its interaction with client polypeptides [78]. The ATP-binding site of HSP70 resides in the N-terminal ATPase domain and has low affinity for client substrate proteins. Currently, two isoforms of DNAJ directly interact with HSP90: DNAJC7 (Tpr2) and DNAJB2 isoform b (HSJ1b) [79]. DNAJC7 contains various highly conserved tetratricopeptide repeat (TPR) motifs that connect other co-chaperones to HSP90 and HSP70, such as HSP90–HSP70 sorting-out proteins (HOP) [80]. The tetratricopeptide-repeat-containing (TPR-containing) chaperone is associated with HSP70 and HSP90 through the C-terminal EEVD motif [80,81].

Another principal function of HSP40 is translocation. For instance, DNAJC6/auxilin and DNAJC26/auxilin-2 play important roles in endocytosis by uncoating clathrin-coated vesicles in association with HSC70 [82]. DNAJs/HSP40s also play a vital role in targeting denatured and damaged proteins for proteasomal degradation in the cytosol by preventing conglomeration.

#### 3.2.2. Role of HSP40 in Carcinogenesis

The overexpression of HSP40 plays a potential role in malignancy progression in many human cancers, such as liver, cervical, gastric, lung, prostate, and colorectal cancers. The dual roles of HSP40 family members are due to their contribution to both pro-malignant and anti-malignant effects [83]. DNAJA3, recognized as Tid1, binds to HSP70 and directly attaches to p53. This interaction results in the translocation of mitochondria, triggering a crucial apoptotic process in MCF-7 breast cancer cells. It functions as a regulator of apoptosis mediated by p53 [84,85]. Decreased Tid1 levels facilitate the promotion of cell growth and a significant reduction in apoptosis in osteosarcoma cells, contributing to Tid1’s role as a tumor suppressor. Conversely, an increase in Tid1 expression is associated with the initiation of colorectal cancer in certain cases [86].

DNAJB11 (ERdj3) is a stress-inducible endoplasmic reticulum (ER) DNAJ homolog that binds to the Kaposi sarcoma-related herpes virus (KSHV) K1 protein, enhances its expression, and shows anti-apoptotic function that promotes cancer [87]. Different members of the HSP40 family exhibit oncogenic activities. The expression of DNAJB1 destabilizes PDCD5 (programmed cell death protein) to inhibit p53-associated apoptosis in multiple cancer cells [88]. The overexpression of DNAJC6 and DNAJB8 has been reported in the development of tumors in hepatocellular carcinoma and renal cancer cells, respectively [89,90].

#### 3.2.3. HSP40 as a Therapeutic Target

HSP40 members regulate the effects of chemotherapeutic agents. An immunological methodology to target HSP40 involves immunization against the DNAJB8 isoform. DNAJ (HSP40) homolog subfamily DNAJB8 belongs to the HSP40 family, and its expression was detected in the development and metastasis of cancer. Enhanced expression of DNAJB8 has also been observed in stem cells derived from cancer (CSCs) in patients diagnosed with renal cell carcinoma. This suggests that DNAJB8 plays a special role in the onset of malignant growth and can be used to target CSCs for therapy [89,91].

In the pursuit of diverse strategies to combat cancer, a range of inhibitors have surfaced, each uniquely targeting and influencing the mechanisms associated with HSP40. N-formyl-3,4-methylenedioxy-benzylidene-γ-butyrolactam (KNK437), a pan-HSP inhibitor, inhibits the expression of various HSPs, including HSP40, in human colon carcinoma cells [92,93]. BMS-690514 is a potent inhibitor of the epidermal growth factor (EGF) receptor (HER)/vascular endothelial growth factor (VEGFR) receptor and exerts anti-apoptotic effects by decreasing the expression of HSP40 and other HSPs in non-small-cell lung cancer (NSCLC) cells [94,95]. Farnesyltransferase inhibitor (FTI) R115777 (tipifamib) is an active inhibitor that restricts tumor development, cell survival, and angiogenesis (HDJ2, VEGF, and MMP1) pathways in breast carcinoma cells [96,97]. In addition, R115777 inhibitor targets HDJ-2 to enhance the radiosensitization of glioblastoma multiforme (GBM) cells [98]. A derivative of phenoxy-N-arylacetamides is known to inhibit the expression of HSP40 but is still in the initial stages of development [99].

Knockdown of DNAJB1 increased the effect of gefitinib (a selective EGF receptor inhibitor) by stimulating mitogen-inducible gene 6 (MIG6) in lung adenocarcinoma A549 human cells [100]. Prenylation is a post-translational modification of type 1 DNAJs/HSP40s. In vivo studies have recently demonstrated that simvastatin treatment inhibits geranylgeranylation in murine mononuclear cells (MNCs) from patients with acute myeloid leukemia (AML) [101]. Enhanced chemosensitivity of AML cells following simvastatin treatment was also shown. It was concluded that simvastatin caused increased chemosensitization in a subset of AML patients by inhibiting the prenylation of DNAJs/HSP-40s [102].

### 3.3. Role of HSP60 in Cancer and Its Potential Therapeutic Target

#### 3.3.1. Structure and Function of HSP60

The molecular chaperone HSP60 has emerged as a potential target in cancer therapy. It is widely distributed in the mitochondria and assists in the correct folding of proteins. HSP60 and its co-chaperone HSP10 form a complex that mediates the folding of proteins imported into mitochondria [103]. Human HSP60 and its bacterial homolog are found mainly in the form of a tetradecameric structure and a homo-oligomeric ring of seven subunits [104]. The human HSP60 protein has three cysteine residues (Cys237, Cys442, and Cys447) but not in the bacterial homolog (GroEL) [105]. HSP60 has three structural domains: equatorial (ATP binding), intermediate (connecting the equatorial and apical domains), and apical (substrate binding) [106].

In addition to mitochondrial protein folding, some evidence indicates that intracellular HSP60 interacts with survivin, mortalin (mitochondrial HSP70), and p53 to mediate apoptosis [107,108]. Thus, increasing the expression of surface HSP60s activates cellular immunity and dendritic cell maturation; consequently, an antitumor response of T-cells is created [109]. Extracellular HSP60 can engage with multiple cell-surface receptors, such as CD14, CD40, and Toll-like receptors (TLRs), causing either a pro- or an inflammatory response [110,111] and presenting tumor-associated peptide antigens to the immune system. The pro-inflammatory effect of HSP60, including cytokines secreted by APCs, induces T-cell activation [112]. In addition, HSP60 induces maturation and activation of dendritic cells through the TLR-2 and TLR-4 signal-transduction pathways, synergizing the proinflammatory action of IFNγ [113].

#### 3.3.2. HSP60 and Cancer Development

HSP60 promotes the survival and progression of certain types of cancer cells. For instance, cytosolic HSP60 prevents the activity of IkB kinase (IKK), resulting in the survival of human cervical carcinoma (HeLa) cells [114]. Other studies have shown that HSP60 stabilizes the mitochondrial pool of survivin [115]. The complete exclusion of HSP60 leads to mitochondrial dysfunction, which in turn triggers apoptosis. This process involves the disintegration of the HSP60 and p53 complex, which results in the stabilization of p53, upregulation of BAX expression, and onset of apoptosis dependent on BAX in breast and colon carcinoma cells [108]. HSP60 regulates apoptosis by interacting with cyclophilin D (a protein within the mitochondrial permeability transition pore). Furthermore, the activation of mitochondrial HSP60 triggers CypD-dependent mitochondrial permeability transition, caspase-dependent apoptosis, and the suppression of tumor growth [116]. HSP60 directly interacts with β-catenin and promotes metastasis of malignant cells [117]. In both early and advanced prostate tumors, there is a considerable increase in the expression of HSP10 and HSP60 [118].

#### 3.3.3. HSP60 as Therapeutic Target

HSP60 plays a vital role in the persistence, advancement, and development of resistance in cancer cells, cytoprotection against cell stressors, and anti-apoptotic potential [119]. For example, 5-FU-resistant SW480 colorectal cancer (CRC) cells showed a reversal of drug resistance after the inhibition of HSP-60 expression [120]. Mizoribine, an imidazole nucleoside antibiotic isolated from *Eupenicillium brefeldianum* derivative, was the first HSP60 inhibitor to be reported. On associating with HSP-60, Mizoribine suppresses its ATPase activity and destroys the HSP60-HSP10 complex [121]. The antitumor effects of bortezomib (a proteasome inhibitor) elevated the expression of HSP60 and HSP90 on cancer cell surfaces and promoted phagocytosis by dendritic cells (DCs) in a murine model of ovarian cancer [122]. Synthetic HSP60 inhibitors, such as o-carboranyl-phenoxy-acetanilide and gold (III) porphyrin complexes, have been found to be highly effective against malignant growth in numerous cancer cell lines [123,124]. Small interfering RNA (siRNA) used to silence the HSP60 gene results in mitochondrial damage, disruption of the Hsp60-p53 complex, Bax expression, and Bax-dependent (caspase-dependent) apoptosis in breast and colon cancers [125]. siRNA-mediated gene silencing of HSP60 in healthy cells does not induce apoptosis [125]. Hence, this approach could be used as a target-specific therapy for breast and colon cancer.

### 3.4. The Role of HSP70 in Cancer Development and Its Application as a Therapeutic Target

#### 3.4.1. Molecular Structure and Cellular Functions of HSP70

**Figure 4:**
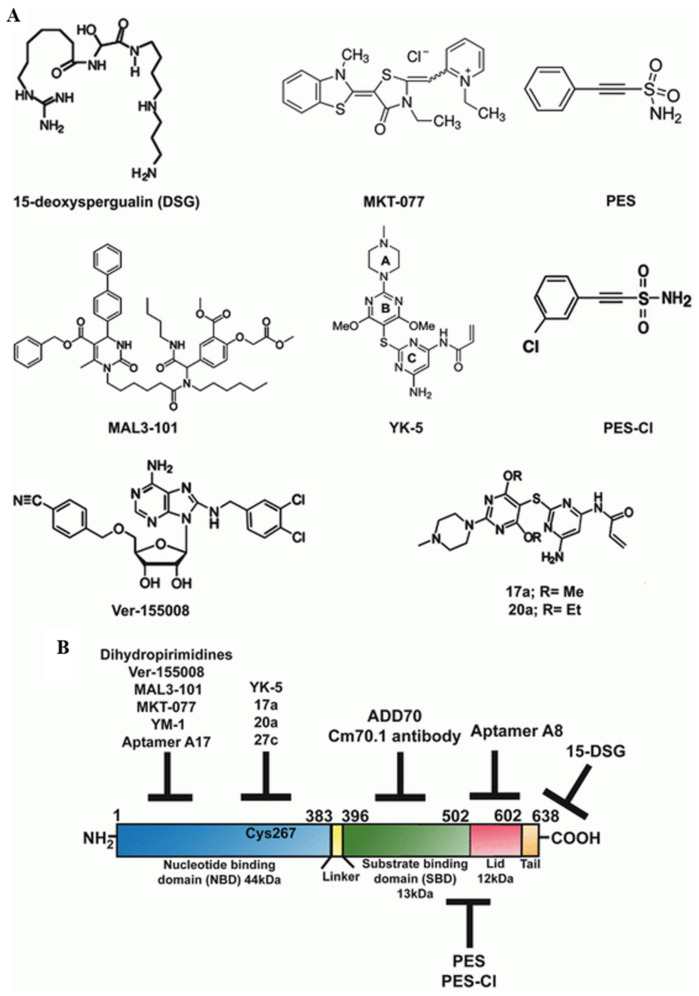
(**A**) Structures of HSP70 inhibitors. (**B**) Schematic diagram of the domain architecture of HSP70 and the possible sites targeted by various inhibitors. Reprinted (adapted) with permission from Ref. [135]. Copyright 2015, Springer Nature.

Human HSP70s comprise a family of 13 members that perform their functions depending on their cellular localization, among which five members that are most concerned with cancer development are stress-inducible HSP70 (HSP72 or HSPA1) and HSPA6 (HSP70B) and constitutively expressed GRP78 (HSPA5), HSC70 (HSPA8), and mortalin (HSPA9) [11]. Under healthy conditions, HSP70 uses ATP to assist in the folding and assembly of newly synthesized polypeptides, maintain proteostasis, and enhance the survival of cells under or after stress [126]. HSP70 contains an interdomain linker that links two distinct highly conserved functional domain structures, namely an N-terminal ATPase domain (45 kDa nucleotide-binding domain, NBD) and a C-terminal polypeptide-binding domain (25 kDa protein substrate-binding domain, SBD) [127]. Furthermore, a short, highly conserved linker links these two domains, which is only 12 residues long, as shown in Figure 4B [127]. Amongst the HSP70 family members, the least conserved region is the C-terminal to the “lid,” which terminates with an EEVD (amino acid designation) motif and is able to interact with the tetra-trico-peptide repeat (TPR) domain. The interaction and sequence variation of HSP70 is believed to allow family members to have distinct co-chaperones, thereby regulating its chaperone function. The co-chaperones of HSP70 are HSP40, Bcl-2-associated athanogene1 (BAG-1), and the C-terminus of HSP70 interacting protein (CHIP).

These can be classified into three categories. They are as follows:

1. The J-domain co-chaperones, a family of HSP40, bind to the NDB of HSP70 and stimulate the ATPase activity of this protein.
2. Nucleotide exchange factor (NEF) co-chaperone members including Bag-1, HSP110, and HSPBP1 (HSP70 binding protein1) stimulate ADP release and complete the chaperone cycle.
3. The tetratricopeptide repeat (TPR) domain co-chaperones members include Hop and CHIP binding to the C-terminal EEVD motif present in both HSP70 and HSP90 chaperones, which is essential for the assembly and intermolecular properties of the HSP70/HSP90 multichaperone complex. Thus, CHIP with ubiquitin ligase activity has also been implicated in the ubiquitination and degradation of HSP70 client proteins [128].

#### 3.4.2. Role of HSP70 in Cancer Development and Survival

HSP70 plays a crucial role in occurrence, progression, and cancer metastasis and is frequently expressed at extreme levels, especially the stress-inducible isoform of HSP70 (also known as HSP-701A1, HSP70-1, or HSP72). Usually, these are undetectable in cells; however, HSP70 can be detected in cells using flow cytometry or confocal microscopy, and their elevated expression helps cancer cells rebalance proteostasis under high-stress conditions [126]. HSP70 overexpression in cancer is associated with the promotion of metastasis, differentiation, apoptosis, tumor growth, drug resistance, and poor prognosis [129]. HSP70 is also involved in most molecular mechanisms, and several checkpoints are involved in cancer development. Evidently, HSP70 is thought to inhibit both the intrinsic and extrinsic apoptotic pathways. In the intrinsic pathway, HSP70 inhibits Bax activation, thereby preventing the permeabilization of the mitochondrial outer membrane and the release of apoptosis-inducing factors [130]. In the extrinsic pathway, HSP70 blocks death-inducing signaling complex (DISC) assembly [131]. Senescence can also act as a powerful antitumor mechanism by preventing the proliferation of cancer cells. Reduced HSP72 expression triggers cell senescence in specific cancer cell lines through pathways leading to senescence that involve both p53-dependent and p53-independent mechanisms [132]. Bag3, serving as a nucleotide exchange factor for HSP-70, is regulated by different transcription factors such as Hif1α, NF-κB, and FoxM1; translation regulators such as HuR; and cell-cycle regulators such as p21 and survivin [133]. Furthermore, BAG3 is also involved in autophagy and cell death. Additionally, HSP70 plays a crucial role in maintaining the genomic integrity of DNA by binding to poly (ADP-ribose) polymerase-1 (PARP-1) in the base pair excision system. DNA-damaging agents have been used extensively in cancer chemotherapy. Hence, the use of HSP70 inhibitors alone or in combination with other drugs may be a better therapeutic strategy for cancer as shown in Figure 4 [134].

#### 3.4.3. HSP70 as an Emerging Drug Target

Three distinct strategies for cancer therapy based on HSP70 are shown in Figure 4:

1. Targeting C-terminal peptide or substrate-binding domain (SBD).
2. Targeting the N-terminal ATP-binding domain (ABD) or N-terminal domain of the nucleotide.
3. Targeting HSP70 co-chaperones.

#### 3.4.4. Targeting C-Terminal Peptide or Substrate-Binding Domain (SBD)

HSP70 has a prominent cytoprotective ability to inhibit apoptosis by neutralizing its function and interacting with apoptosis-inducing factor (AIF) and apoptotic protease activating factor-1 (APAF-1) [136]. Garrido and co-workers rationally designed decay targets of HSP70, which are derived from AIF that neutralized HSP70 function, and hence inducted the sensitization of cancer cells and apoptosis [137]. The blocking peptide derived from AIF corresponds to amino acids 150–228 in the AIF region, which is a crucial segment for binding to the SBD of HSP70, referred to as peptide ADD70 (AIF-Derived Decay). ADD70 disrupts the interaction between HSP70 and AIF, along with other proteins designated as client proteins [137]. 2-phenylethynesulfonamide (PES), a small-molecule inhibitor of HSP70, specifically interacts with its C-terminal substrate-binding domain of HSP70 (Figure 4). Consequently, this triggers the aggregation of improperly folded proteins, disrupts autophagic processes, and destabilizes the usual functions of lysozyme, ultimately prompting autophagic cell death [138]. The outcome of PES induction of either autophagic or apoptotic cell death is contingent on the cell type.

#### 3.4.5. Targeting the N-Terminal ATPase Domain (ABD) or N-Terminal Nucleotide-Binding Domain (NBD)

Compounds that replace ATP from HSP70 are anticipated to be powerful tools for regulating HSP90 inhibitors. VER-155008 an adenosine-based HSP70 inhibitor binds to the ATPase site of the HSC70/BAG-1 crystal structure. It has also been reported that VER-155008 targets the degradation of various HSP90 client proteins such as CDK4, HER2, and RAF-1 [139]. Interestingly, MKT-077 is a cationic rhodacyanine dye analog that binds to a negatively charged pocket close to the ABD of HSP70 and thus disrupts the HSP70 folding cycle [140]. MKT-077 has been explored as an anticancer agent in mouse xenografts, and the trial showed severe renal impairment and toxicity, thus limiting further study of MKT-077 [141]. Recently, YM-1, a derivative of MKT-077, was shown to exhibit selective toxicity against diverse tumor cell lines, highlighting its potential as a promising therapeutic candidate [142]. Reports indicate that VER-155008 is effective in disrupting the degradation of several HSP90 client proteins, including CDK4, HER2, and RAF-1 [35]. Notably, MKT-077, a cationic rhodacyanine dye analog, binds to a negatively charged pocket near the ABD of HSP70, thereby disrupting its folding cycle of HSP70.

Screening for HSP70 ATPase activity revealed that 15-deoxyspergualin (DSG) inhibits HSP70 steady-state ATPase activity by interacting with the C-terminus and hindering its function [143]. However, the efficacy of DSG as an anticancer drug has been limited, which has subsequently led to the search for compounds with structural similarity, resulting in the identification of MAL3-101, a second-generation dihydropyrimidine [144]. MAL3-101 inhibits the ability of HSP40 co-chaperone, accelerates the ATPase activity of HSP70, and prevents its function [145]. Recently, smaller peptide aptamers, A17, were developed using peptide aptamer libraries of *Escherichia coli* thioredoxin scaffolds. A17 binds to the ATP-binding domains of HSP70 and specifically inhibits chaperone activity [146].

Multhoff and Hightower reported that the presence of HSP70 on the surface of tumor cells stabilizes the lysosomal membrane of tumor cells to escape from cell death as a potential therapeutic target [147,148]. HSP70 is expressed only on the surface of tumor cells and stains positively for the IgG1 mouse monoclonal antibody (mAb) cmHSP70.1 [149]. Furthermore, mAb CmHSP70.1 recognizes conserved epitopes in the C-terminal amino acids (aa 450–461) of inducible HSP70 on both viable human tumors (in *vitro*) and mouse tumors (in *vivo*), as shown in Figure 4B [150]. The injection of the Cm70.1 antibody into mice-bearing tumors significantly inhibited tumor growth and improved overall survival. This finding indicated that cmHSP70.1 could be used as a therapeutic agent.

#### 3.4.6. Targeting HSP70 Co-Chaperones

The best way to inhibit HSP70 function involves determining specific molecules that precisely interrupt the interaction between HSP70 and its specific co-chaperone; however, this has not been explored in detail. Using a high-throughput screening approach, pyrimidotriazinediones were designed to interfere with Hop/HSP70 interaction and were found to be toxic to WST-1 cells in vitro [151]. It was also recently reported that targeting Hip impairs the proteostatic activity of the HSP70 chaperone and stimulates neurodegeneration [152].

### 3.5. HSP90 as a Target for Cancer Development

HSP90 is a vital chaperone and client protein of the HSPs family that is involved in cancer progression. HSP90 is the most abundant, highly conserved, and expressed molecular chaperone in eukaryotic cells, representing 1–2% of the total cellular proteome under physiological homeostatic conditions. Under stress, HSP90 expression rises to approximately 4–6% of cellular proteins, stabilizing itself and boosting cell survival [153]. HSP90 is essential for promoting the development, resilience, and effectiveness of cancer-causing client proteins [154]. HSP90 performs housekeeping functions such as proper folding of newly synthesized proteins, restoration of denatured proteins to their functional states, directing proteins to specific cellular compartments, averting the aggregation of proteins, facilitating the maturation of signaling proteins, and orchestrating the regulated degradation of proteins [155]. HSP90 functions in a multicomponent machine of chaperone proteins, including p23, p60/Hop, p50Cdc37, HSP40/HDJ2, HSP70, and various immunophilins [156]. HSP90 is unique among molecular chaperones due to its preference for protein kinases and steroid hormone receptors as client proteins. These proteins play essential roles in signal transduction pathways [156].

Many of these client proteins, including erythroblastic oncogene B (ERBB2), tumor suppressor protein (p53), breakpoint cluster region-Abelson (Bcr-Abl), epidermal growth factor receptor 2 (HER2), B-Raf proto-oncogene serine/threonine kinase (B-Raf), C-Raf proto-oncogene serine/threonine kinase (C-Raf), protein kinase B (AKT), mesenchymal-epithelial transition factor (MET), vascular endothelial growth factor (VEGF), fms-like tyrosine kinase 3 (FLT3), hypoxia-inducible factor (HIF)-1, telomerase, androgen, and estrogen receptors, have been shown to promote cell growth, proliferation, and cell survival. Many of these clients are frequently mutated and/or overexpressed in cancer cells [157,158,159]. HSP90 frequently exhibits higher levels of expression in comparison to its healthy counterparts, and it plays a vital role in promoting the proliferation and survival of cancer cells. Additionally, there have been reports indicating that the suppression of HSP90 results in the breakdown of client proteins through the ubiquitin/proteasome pathway. HSP90 is essential for correctly folding client proteins and has become an attractive target for the development of anticancer medications [159].

#### 3.5.1. Structure and Functional Relationship of HSP90

HSP90 is a large and flexible homodimer protein that functions under physiological conditions. Each monomeric unit is composed of three highly conserved functional domains: the N-terminal domain (NTD) or ATP-binding domain, the middle domain (MD) or catalytic domain, and the C-terminal domain (CTD) or dimerization domain [160]. In addition, it has specific sites for its binding partners, including co-chaperones and client proteins. The N-terminal domain contains a nucleotide-binding pocket for binding to ATP/ADP. Hence, the binding of ATP and its subsequent hydrolysis provide the energy required for protein folding. The C-terminal domain provides dimerization for HSP90 and has a putative ATP-binding pocket that serves as an allosteric regulator for the function of HSP90. The middle domain provides a site for the interactions of client proteins and co-chaperones with HSP90 to form the HSP90–co-chaperone–client protein complex. Hence, the dynamic nature of the HSP90 chaperone cycle provides multiple targeting sites for the modulation of chaperone activity using small-molecule ligands. Moreover, some members of the HSP90 family, such as cytosolic eukaryotic HSP90 and GRP94 (94 kDa glucose-regulated protein), have disordered regions, termed the charged linker, which separates NTD and MD. Furthermore, cytosolic eukaryotic HSP90 contains an MEEVD motif at the C-terminal extension. The NTD- or ATP-binding domain of HSP90 is unique and distinct from the ATP-binding cleft of HSP70 and other protein kinases. The p23 (yeast homolog Sba1) and p50 (yeast homolog Cdc37) co-chaperones are also connected to the NTD binding site [161].

**Figure 5:**
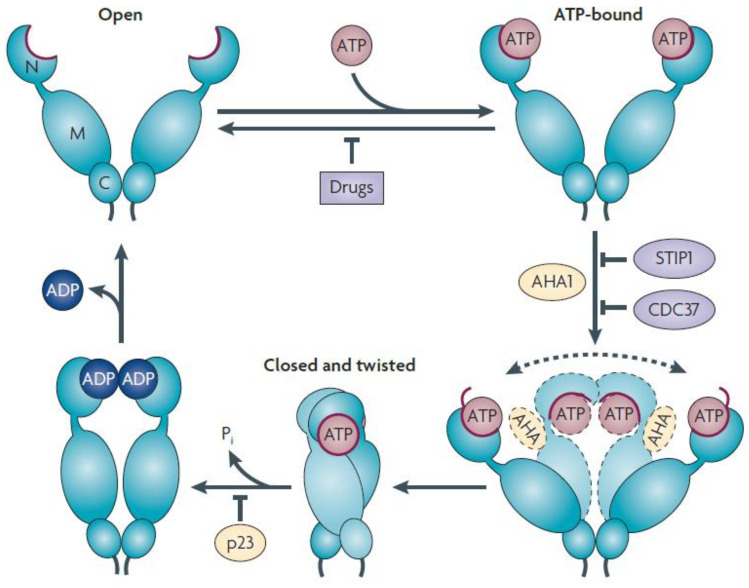
The chaperone, HSP90, undergoes multiple conformational states in the absence of nucleotides or other factors. ATP binding and hydrolysis shift conformational equilibrium by lowering the energy barrier between specific conformations, thus providing a conformational cycle for HSP90 [165]. ATP binds to the undimerized NTD (open state) of HSP90 and leads to N-terminal dimerization caused by the “lid” segment (red) repositioning. The subsequent structural rearrangement establishes a (closed and twisted) conformation of HSP90, which is suitable for ATP hydrolysis. The co-chaperone AHA1 increases HSP90 ATPase 1 activity by promoting conformational changes required to achieve ATPase competence. In the absence of AHA1, it was very difficult for HSP90 to achieve ATPase-competent conformation (dotted arrow). A number of co-chaperones, including Sti1p (p60HOP), Cdc37 homolog (cell division cycle 37), and N-domain-binding HSP90 inhibitors, prevent dimerization of the N-terminal domain. PTGES3 (prostaglandin E synthase 3 cytosolic, so-called p23 protein) deregulates the ATPase cycle and stabilizes its closed conformation. Reprinted (adapted) with permission from Ref. [163]. Copyright 2010, Springer Nature.

The MD of HSP90 comprises a binding site for co-chaperone Aha1 (activator of HSP90 ATPase activity1) and client proteins such as Cdk4, Akt, eNOS, phycobilisome-linker polypeptides, and staphylococcal nuclease. The dimerization of HSP90 is crucially influenced by its carboxy-terminal domain, which is also responsible for interacting with client proteins such as the tumor suppressor protein p53 [162]. The N-terminal ATP binding site leads to the dimerization of HSP90 (open state) and facilitates co-chaperone and client protein binding. The ATP hydrolysis conformational cycle of HSP90 (closed state) was enhanced by the co-chaperone, as shown in Figure 5 [163]. Several co-chaperones, Hop (HSP organizing protein-yeast homolog Sti1), protein phosphatase 5 (PP5-Yeast homolog Ppt1), and FK506-binding protein FKBP52, which contains multiple TPR domains, have the ability to recruit the HSP90 chaperone and simultaneously bind to the C-terminal MEEVD motif of HSP90 [164].

#### 3.5.2. Targeting the N-Terminal Nucleotide Binding Domain

The nucleotide-binding pocket found in the N-terminal domain (NTD) of HSP90 binds with ATP, and this unique pocket structure is a common feature within the GHKL (gyrase, HSP90, histidine, kinase, MutL) superfamily of proteins. These compounds prevent ATP binding and hydrolysis, leading to the proteasomal degradation of proteins assisted by HSP90. Natural inhibitors, such as radicicol (RD) and geldanamycin (GM), hinder HSP90 by interacting with the N-terminal site, a structural mimicry of ATP [166]. Although these natural HSP90 inhibitors exhibit potential antitumor activities, their clinical research and applications are limited owing to their poor solubility and significant hepatotoxicity [128], which has led to the development of second-generation HSP90 inhibitors using derivatives of natural inhibitors. To improve solubility and reduce toxic side effects, semi-synthetic derivatives of GM, including IPI-504, 17-AAG, and 17-DMAG, have been developed for better clinical features. RD and its derivatives NVP-AUY922, AT-13387, STA-9090 (ganetespib), and KW-2478 have been developed with improved stability and clinical properties. Among the derivatives of GM, IPI-504 was identified as a prodrug of 17-AAG, specifically a hydroquinone hydrochloride formulation, exhibiting enhanced potency and improved toxicity profiles in preclinical investigations compared to its parent compound. Results from Phase 1 clinical trials indicated that KW-2478, used for multiple myeloma either independently or along with bortezomib, exhibited a positive response rate of 39% and a progression-free survival of 26.4 weeks [167]. The availability of X-ray crystallographic structures for HSP90 attached to ATP, geldanamycin, and radicicol has proved pivotal in formulating HSP90 inhibitors, ultimately contributing to the development of synthetic inhibitors. The first purine-based synthetic HSP90 inhibitor, PU3, was identified by Tony Taldone and Gabriela Chiosis [168], along with other inhibitors synthesized later as a common pharmacophore. In addition, other purine analogs that inhibit HSP90 include PU-H71, MPC-3100, Debio 0932/CUDC-305, and CNF2024/BIIB021, which have been synthesized by various research groups.

#### 3.5.3. Targeting the HSP90 C-Terminal Binding Protein

A novel molecular strategy for regulating HSP90 chaperone activity has emerged through the introduction of a fresh ATP-binding site at the C-terminus. This addition induces the modulation of allosteric interactions between the C-terminus and N-terminus of HSP90, presenting a unique approach to controlling its chaperone function [169]. Although most HSP90 inhibitors bind/target the N-terminal ATP-binding site of HSP90, the C-terminus can also be targeted by the well-known natural products of aminocoumarin antibiotics, such as novobiocin, chlorobiocin, and coumermycin A inhibitors of HSP90 [163]. Novobiocin, isolated from *Staphylococcus saprophyticus*, is a known DNA gyrase inhibitor that inhibits both HSP90 and DNA gyrase B, belonging to the GHKL family [170]. Several synthetic C-terminus HSP90 inhibitors such as Epigallocatechin-3-gallate (EGCG), cisplatin, molybdate, taxol [171,172,173,174], and withaferin A have been identified. The majority of HSP90 inhibitors, both N-terminal HSP90 ATPase inhibitors and C-terminal ATPase inhibitors, are listed in Table 2.

#### 3.5.4. Targeting Co-Chaperones and Their Interaction with HSP90

The co-chaperone Cdc37 promotes the connection of HSP90 with the kinase protein subset of client proteins to maintain their stability and signaling functions. Interrupting the complex between Cdc37 and HSP90 has surfaced as an alternative focal point for cancer progression. In human colon cancer cells, the silencing of Cdc37 destabilizes various kinase clients (AKT, HER2, CDK4, CDK6, and CRAF) and sensitizes cancer cells to HSP90 inhibitors and apoptosis [179]. Another study reported that the selective inhibitors celastrol and gedunin inactivated the co-chaperone p23 and affected HSP90 function through multiple mechanisms [180,181]. Targeting the interplay between HSP90 and Aha1 offers an alternate approach to modulating HSP90 activity. Aha1, which functions as an activator of HSP90 ATPase, forms associations with the N-terminal and middle domains of HSP90, thereby enhancing the ATPase activity of HSP90. Silencing Aha1 in colon cancer cells through siRNA resulted in a reduction in the activity of Raf-1 kinase and a decrease in the phosphorylation of MEK1/2 kinase and ERK1/2 kinase [182]. In PC3-MM2 prostate cancer cells, HSP90 C-terminal inhibitors, such as KU-135 and novobiocin, disrupted the HSP90α/Aha1 complex, leading to inhibited cell migration, which is a critical factor in tumor metastasis [183].

#### 3.5.5. Targeting HSP90–Client Protein Interactions

Another alternative strategy for inhibiting HSP90 chaperone activity is to target interactions between HSP90 and its client proteins. Androgens drive prostate cancer cell growth through the androgen receptor (AR), which depends on HSP90 for survival and progression. In prostate cancer, HSP90 regulates the activity and stability of the AR by forming the HSP90-AR complex [184]. Nevertheless, disruption of this complex impedes the nuclear translocation of AR, induces cytosolic aggregation, and ultimately leads to AR degradation [185]. Camptothecin, a DNA topoisomerase 1 inhibitor that disrupts the HSP90-AR complex, inhibits AR transcriptional activity and suppresses prostate cancer cell growth [186]. Recent studies have demonstrated that HSP90 interacts with survivin, an inhibitor of apoptosis. However, dissociation or disruption of the HSP90–survivin complex leads to survivin degradation, suppresses cell proliferation, and inhibits mitochondrial apoptosis [158]. Plescia et al. demonstrated that shepherdin, a cell-permeable peptidomimetic compound, is capable of disrupting the HSP90–survivin complex and exhibits the loss of various client proteins, including CDK4, CDK6, Akt, and survival. Conversely, treatment of MCF-7 xenograft models with shepherdin resulted in a nearly complete loss of Akt levels in tumor cells [187].

### 3.6. Large HSPs and Cancer

The two main large HSPs HSP110 and GRP170 are relatively conserved and distinct sets of stress proteins, whose expression is induced by heat shock and glucose starvation, respectively. Under normal circumstances, the cytoprotective nature of HSP110 prevents stress-induced apoptosis in neuronal cells [188]. The role of HSP110 in apoptosis inhibition by genetically silenced HSP110 in various types of cancer cells that induce apoptosis has been previously reported [189]. HSP110 hinders the release of cytochrome c, a mitochondrial protein, thereby blocking caspase activation and preventing caspase 3- and 9-mediated apoptosis [188,189]. GRP170 is another high-molecular-weight family of HSPs that has been less studied than other conventional HSPs about its role in cancer prognosis and diagnosis. The immune adjuvant properties of GRP170 are gaining importance in cancer therapy owing to its intrinsic ability to induce both innate and adaptive immune responses [190,191].

#### Large HSPs as a Therapeutic Target

Elevated levels of HSP110 have been detected in numerous cancer types, suggesting that it is a potential therapeutic target for various cancers, including melanoma, colorectal cancer, and non-Hodgkin lymphoma [192,193]. The immunogenic characteristics of large HSPs present an opportunity to develop novel cancer vaccines. In a cervical cancer mouse model, simultaneous administration of the HSP110-E7 epitope complex and T lymphocyte epitope E7 exhibited improved efficacy against tumors. This combined approach resulted in decreased tumor growth and prolonged survival compared with other interventions [194]. Moreover, vaccination with HSP110 yielded favorable outcomes in mouse models of intestinal adenomas and spontaneously developed breast tumors [195]. GRP170 has also been used as a vaccine for the development of potential cancer antigens. Co-administration of specific tumor protein antigens along with the GRP170 complex resulted in a better immune response in the melanoma model [196]. In the first phase of the clinical trial, subjects diagnosed with third- and fourth-stage melanoma were enrolled to assess the efficacy of a vaccine incorporating the chaperone complex comprising human HSP110 and gp100, which had previously demonstrated success in preclinical evaluations (NCT01744171).

### 3.7. HSF1 as a Potential Cancer Therapeutic Target and Biomarker

HSF1 is a key regulator of the heat shock response (HSR), a mechanism that is retained by addressing proteotoxic stress. This process ensures the timely expression of HSPs, promoting cell viability through enhanced proteostasis [197]. Notably, HSF1 involvement extends to cancer, influencing migration, invasion, and metastasis [198,199]. Its overexpression has been observed in human prostate cancer, particularly in malignant epithelial cells, suggesting that HSF1 is a potential biomarker for various cancer types [200]. Hoang et al. revealed HSF1 upregulation in malignant prostate epithelial cells compared to normal prostate cells in primary cancer specimens [201].

In several malignancies, increased levels of HSF1 are associated with an unfavorable prognosis. Elevated nuclear levels of HSF1 have been linked to increased tumor growth, lymph node invasion, and death in cases of breast cancer, particularly in individuals who test positive for the estrogen receptor (ER) [202]. Similar associations with poor overall and relapse-free survival have been observed in breast cancer patients with high HSF1 mRNA expression. HSF1 also serves as a prognostic biomarker in endometrial cancer, hepatocellular carcinoma (HCC), esophageal carcinomas, and oral squamous cell carcinoma (OSCC) [200,203]. The overexpression of HSF1 has been linked to aggressive disease progression and reduced survival in these cancer types. Additionally, phosphorylated HSF1, specifically phospho-Ser326, has been identified as a potential marker for HSF1 activity and is associated with poor prognosis in ovarian cancer [204]. Collectively, these findings underscore the significance of HSF1 expression as a valuable prognostic indicator in diverse cancers.

Inhibiting transcription factors, such as HSF1, is challenging because of their complex tertiary structures and lack of target sites. HSF1, which is a ligand-less factor with poor druggability, poses difficulties in achieving inhibition specificity. Genetic approaches, such as RNA silencing, have been extensively pursued to overcome this challenge. Currently, both genetic and pharmacological strategies are being explored to inhibit HSF1 [205].

HSF1 regulates cellular stress responses, including HSP expression. The compound KRIBB11 inhibits HSF1 activity by preventing the binding of P-TEFb to the promoter of the HSP70 gene, thus reducing the transcription of stress response genes [206]. In animal models, KRIBB11 demonstrated HSF1 inhibitory effects, lowering HSP70 expression and tumor volume. KRIBB11 successfully prevented lymphatic metastasis in bladder cancer without causing any noticeable toxic effects. When combined with an AURKA inhibitor (danusertib), KRIBB11 exhibited strong anticancer activity against liver cancer by inducing cancer cell apoptosis and triggering an ER stress response [207]. Nonetheless, some studies have reported inconsistent results, indicating that KRIBB11 may not directly inhibit HSF1 in certain contexts, such as ferroptosis-related processes involving PROM2 [208].

Triptolide, a component of *Tripterygium wilfordii*, acts as an HSF1 inhibitor. In chronic lymphocytic leukemia (CLL), where HSF1 is overexpressed, triptolide causes apoptosis in both primary and cultured CLL B cells. When HSF1 is inhibited by knockdown or triptolide, it reduces HSP90 levels and impairs the cytosolic complex, which consists of deacetylase-histone deacetylase 6 (HDAC6), HSP90, p97, HSF1, and p97. Accordingly, HSF1 inhibition leads to the acetylation of HSP90, which disrupts the function of chaperones and suppresses malignancy in CLL [209].

DTHIB (SISU-102), a synthetic compound identified by screening for interactions with the HSF1 DNA-binding domain, selectively targets and inhibits HSF1 by increasing the degradation of HSF1 trimers (active nuclear). This potent inhibition reduces the excess amount of the multichaperone complex. In prostate cancer, DTHIB surpasses enzalutamide in reducing cell viability, inhibiting androgen receptor signaling, and suppressing PSA expression. DTHIB also hinders cell proliferation and diminishes cancer growth without noticeable side effects in mouse models [210]. Notably, in an acute myeloid leukemia model, DTHIB selectively suppresses the self-renewal of leukemia stem cells while leaving normal progenitor/hematopoietic stem cells. This effect is achieved by inhibiting the targets of HSF1, including HSP90, as well as suppressing mitochondrial oxidative phosphorylation by downregulating SDHC, a significant enzyme complex in the TCA cycle [211].

## 4. HSP-Based Therapies for Cancer

### 4.1. HSP-Based Vaccines and Cancer Immunotherapy

HSPs function as chaperones within cells and bind to antigens (peptides) associated with tumors. Moreover, APCs can recognize complexes formed by HSPs and peptides, leading to specific antitumor responses [39]. HSPs play a role in enhancing immune responses during the presentation of tumor antigens. Consequently, there is evidence suggesting that immunization with HSPs obtained from tumor cells can elicit an immune response against the tumor [212]. Various members of the HSP chaperone families, connected with diverse cancer-peptide-based antigens, may be isolated from tumor cells. By purifying the heat-shock-protein–peptide groups/complexes from a patient’s isolated cancer, this complex can serve as a personalized tumor vaccine that delivers antigens derived from tumor cells to the immune system, thereby fostering anticancer immunity [213].

Scientific evidence on the link between HSPs and cancer vaccination has been supported by various studies. Chen et al. recently developed a vaccine composed of fusion proteins such as HSP65 and a peptide (octa) epitope from the six transmembrane epithelial antigens of the prostate 1 (STEAP1) [214]. STEAP1 is highly expressed in multiple types of cancers. The fusion protein HSP65/STEAP1 demonstrated the ability to inhibit or arrest B16F10 melanoma growth (xenograft) and mouse RM-1 prostate cancer [215]. Furthermore, His-HSP65 (HHSP65), a fusion protein, stimulates the expression of TNF-α and facilitates STEAP1 to enhance TNF-α secretion, effectively inhibiting cancer cell proliferation [216]. When combined with IFN-γ, TNF-α triggers the upregulation of MHC-II expression, initiating cellular immune responses and amplifying the cytotoxic activity of various immune cells. Moreover, a vaccine targeting the CD133 epitope coupled with an adjuvant such as gp96 facilitated the transfer of epitope-specific cytotoxic T lymphocytes (CTLs), effectively suppressing leukemia growth in a murine xenograft model [217]. Immunization with bone-marrow-derived dendritic cells derived from bone marrow (BMDCs) stimulated by placental gp96 resulted in reduced tumor growth and enhanced survival in mice. This approach elicits a robust tumor-specific T-cell response, establishing its efficacy as an immunotherapeutic strategy [218].

Recent clinical assessments have broadened the investigation of cancer immunotherapy utilizing HSP-based vaccines. A newly developed self-assembled nanochaperone inspired by HSPs has the potential to significantly augment cancer immunotherapy. This nano-vaccine employs HSP-like microdomains and mannose surface decoration to capture antigens, facilitating their movement into dendritic cells [219]. This process promotes the escape of antigens and improves their cross-presentation in the cytoplasm. This approach has demonstrated its capacity to stimulate responses from the immune system, including both CD8^+^ T cells (cytotoxic cells) and CD4^+^ T cells (helper cells), for the prevention of melanoma [219]. The combination of chaperone protein immunotherapy with checkpoint inhibitors of the immune system, such as anti-PD-1/PD-L1, is particularly emphasized in the treatment of melanoma [220].

Furthermore, clinical trials recorded on ClinicalTrials.gov have assessed the effectiveness of a combined intramuscular (IM) vaccination regimen involving pNGVL4a-Sig/E7(detox)/HSP70 DNA and singular intramuscular immunization with TA-CIN to promote clearance of human papillomavirus 16 (ClinicalTrials.gov Identifier: NCT03911076) [221]. Another set of trials investigated the GP96 HSP-peptide complex vaccine as a potential treatment for patients with liver cancer (ClinicalTrials.gov Identifier: NCT04206254) [222].

Glioblastoma, a primary brain cancer with poor or substantial prognosis, is under investigation for its effectiveness [223]. Standard therapy combined with HSPPC96-based vaccination has shown safety and efficacy in patients diagnosed with glioblastoma. TSIR, known as the tumor-specific immune response, predicts the vaccine’s efficacy, with high TSIR correlating with a median overall survival exceeding 40.5 months [224]. Further studies involve the sequencing of T-cell receptors to analyze the T-cell receptor repertoire in tumor-infiltrating lymphocytes (TILs) and identify potential biomarkers for predicting responses to HSPPC96 vaccination [225]. Additionally, a phase II trial in glioblastoma patients (adults) undergoing surgical resection following standard therapy and autologous HSP peptide vaccine (Prophage) administration revealed MGMT promoter methylation as a prognostic factor, with significantly extended overall median survival for methylated tumors compared to unmethylated tumors [226].

### 4.2. Photothermal and Modulated Electro-Hyperthermia Therapy

Emerging treatments for cancer include photothermal therapy (PTT) and modulated electro-hyperthermia therapy (mEHT). Both approaches to selectively target and eliminate malignant cells involve intentionally raising the temperature, which is referred to as hyperthermia. PTT mostly uses light-absorbing substances, usually nanoparticles. These substances absorb light energy at a certain wavelength and turn it into heat, which raises the temperature in the area, causing localized hyperthermia and damaging or killing cancer cells. Modulated electro-hyperthermia therapy is another form of hyperthermia therapy that involves the application of modulated electric fields to precisely elevate the temperature of cancer cells. Owing to their higher conductivity, cancer cells can consume a larger quantity of energy and experience higher temperatures than normal cells. However, the major problem associated with hyperthermia is the rapid expression of HSPs. However, the main problem with hyperthermia is that it causes rapid production and release of HSPs, which help proteins float inside cells and increase their tolerance [227]. The therapeutic efficacy of PTT is directly influenced by intracellular HSP expression levels, which have both protective and detrimental effects on cancer cells. Thus, the efficacy of hyperthermia therapy can be increased by inhibiting HSPs, which can reduce the thermal resistance of cancer cells and potentiate the cell-killing effect of hyperthermia [228]. Currently, HSP inhibitors are being used in combination with hyperthermia. For instance, Kuo et al. reported that mEHT increased efficiency by combining with the HSP inhibitors nano-curcumin and resveratrol in an in vivo CT26/BALB/c animal tumor model caused by decreased HSP70 expression and the infiltration of immune cells (CD3+ T-cells and F4/80+ macrophages) into tumors receiving this treatment [229].

### 4.3. Role of Chaperone-Mediated Autophagy in Cancer Diseases

Chaperone-mediated autophagy (CMA) primarily focuses on the degradation of misfolded proteins by translocating these polypeptides across the lysosomal membrane, thereby helping to maintain cellular homeostasis. There are reports on the dual nature of CMA in cancer because of its protumorigenic and antitumorigenic potential [230]. In a pro-tumorigenic role, CMA helps to survive and grow cancer cells through the modulation of the cell cycle. Hypoxia is an important characteristic observed in the tumor microenvironment, which helps activate CMA gene transcription. This CMA assists cancer survival and progression in the hypoxic microenvironment of tumors by hypoxia-inducible factor-1 alpha (HIF-1α) degradation (2). Similarly, the anti-oncogenic role or tumor-suppressive potential of CMA has been reported in the tumor microenvironment, where CMA is involved in protooncogene proteins such as Murine Double Minute 2 (MDM2) and tumor-associated translationally controlled tumor protein (TCTP) upon acetylation [231,232]. Although research in the field of CMA is still being conducted to examine the connection between faulty CMA and cancer, the translation of these results into preventative or therapeutic measures has been hampered by several issues. In the initial stage, every component and modulator of CMA, such as signaling elements and chaperones, participates in a wide range of crucial cellular processes. To date, no research has been conducted to identify a particular component of the CMA pathway that may be used as a target for external manipulation. This significant barrier must be overcome to discover selective chemical modulators of CMA. For instance, LAMP2A knockdown by direct injection of shRNA results in tumor regression and reduced metastasis in human lung cancer xenograft mice [233].

## 5. Conclusions

HSPs are a conserved family of proteins that are highly expressed in several types of cancer. Cytoprotective functions are crucial for cancer cell survival. HSPs are often associated with cancer progression and response to treatment, such as drug resistance, as well as the overall poor prognosis of patients. The increased prevalence of HSPs in cancer cells has led to over a decade of research, which has made them a potential target for oncologists to develop targeted inhibitors for small HSPs. These small-HSP inhibitors can alter the cellular expression of HSPs and subsequently their activity, thereby inhibiting the proliferation of malignant cells in cancer therapy. They have also been used as chemosensitizing compounds with different chemotherapeutic agents to overcome acquired drug resistance or can yield synergistic cytotoxic effects in cancer therapy. Although there have been a number of reports on their synthesis and application as inhibitors of cancer cell proliferation, sufficient investigation of animal models and the side effects of these inhibitors have not been well reported. Moreover, the molecular mechanisms of action of various HSPs, especially those associated with cancer progression, are yet to be clearly understood, hindering the development of suitable biocompatible HSP inhibitors in cancer therapy.

## Figures and Tables

**Table 1:** Summary of various families of HSPs and their members with details of cellular location and key functions.

HSP Family	Important Members	Co-Chaperones	Location	Functions	Ref.
Small HSPs	HSP10	None	Mitochondria	Binds with HSP60 to form chaperonin complex, favors cellular protein folding	[20,21]
	HSP27		Cytosol/ Nucleus	Prevents unfolded protein aggregation	
HSP40/DNAJ	HSP40	None	Cytosol	Co-chaperone for HSP70, enhances the rate of its ATP activity and substrate release	[22]
	Tid1	None	Cytosol		
		None	Mitochondria		
HSP60	HSP60	HSP10	Cytosol, mitochondria, chloroplast	Protein folding and assembly with the help of HSP10	[23]
HSP70	HSP70	Bag1, HSP40, Grpe, Hip, Hop, CHIP, Bag3,	Cytosol	Employed in trafficking of proteins, their degradation, and refolding of denatured proteins during stress	[24,25]
	HSP70-2		Cell surface		
	HSC70		Cytosol		
	GRP75/Mortalin		Mitochondria		
	GRP78		Endoplasmic reticulum		
HSP90	HSP90A	P23, FKBP51, FKBP52, dc37, Aha1, Cyp40, Hop	Cytosol	Assist folding, maturation, intracellular transport, and degradation of proteins	[26,27]
	HSP90B		Cytosol		
	GRP94		Endoplasmic reticulum and cytosol		
	TRAP1		Mitochondria		
Large HSPs	HSP110	None	Cytosol	HSP110 regulates the kinetics of HSP70 and substrate interaction in thermal stress condition.	[28,29,30]
	GRP170		Endoplasmic reticulum	Folding assembly and transportation of secretory or transmembrane proteins	[31,32]

**Table 2:** List of commonly used macrocyclic HSP90, N-terminal, and C-terminal ATPase inhibitors.

Site of Action	Class of Inhibitors	Examples	Ref.
N-terminal ATPase	Benzoquinone	Geldanamycin (GDA)	[175]
	Ansamycin	Tanespimycin (17-AAG) Alvespimycin (17-DMAG) Retaspimycin (IPI-504)	[50]
	Macrolide	Radicol (RDC)	[176]
		Radicol-based: Gantespib (STA-9090) CCTO18159 NVP-AUY922 KW-2478 AT13387	
	Chimeric Inhibitors (GDA+RDC)	Radanamycin Radamide Radester	[177]
	Purine Scaffold	PU3	
	Pyrazole	PU24FC1 CCTO18159	
C-terminal ATPase	Coumarin Antibiotics	Novobiocin Novobiocin-based: Cholorobiocin Coumermycin	[165]
	Synthetic Inhibitors	EGCG (Epigallocatechin-3-gallate) Cisplatin, Taxol, Withaferin	[178]

## Data Availability

This paper contains all relevant data; no additional data required.
